# Nephrology for the people: Presidential Address at the 42nd Regional Meeting of the Japanese Society of Nephrology in Okinawa 2012

**DOI:** 10.1007/s10157-013-0776-x

**Published:** 2013-02-08

**Authors:** Kunitoshi Iseki

**Affiliations:** Dialysis Unit, University Hospital of the Ryukyus, 207 Uehara, Nishihara, Okinawa 903-0215 Japan

**Keywords:** Survival, Predictor, Chronic kidney disease (CKD), End-stage kidney disease (ESKD), Proteinuria

## Abstract

The social and economic burdens of dialysis are growing worldwide as the number of patients increases. Dialysis is becoming a heavy burden even in developed countries. Thus, preventing end-stage kidney disease is of the utmost importance. Early detection and treatment is recommended because late referral is common, with most chronic kidney disease (CKD) patients remaining asymptomatic until a late stage. Three-quarters of dialysis patients initiated dialysis therapy within 1 year after referral to the facility. Since its introduction in 2002, the definition of CKD has been widely accepted not only by nephrologists but also by other medical specialties, such as cardiologists and general practitioners. Japan has a long history of general screening for school children, university students, and employees of companies and government offices, with everybody asked to participate. The urine test for proteinuria and hematuria is popular among Japanese people; however, the outcomes have not been well studied. We examined the effects of clinical and laboratory data from several sources on survival of dialysis patients and also predictors of developing dialysis from community-based screening (Okinawa Dialysis Study, OKIDS). At an early CKD stage, patients are usually asymptomatic; therefore, regular health checks using a urine dipstick and serum creatinine are recommended. The intervals for follow-up, however, are debatable due to the cost. CKD is a strong risk factor for developing cardiovascular disease and death and also plays an important role in infection and malignancies, particularly in elderly people. People can live longer with healthy kidneys.

## Introduction

Although kidney disease patients can survive without kidney function, dialysis is a life-saving procedure. However, many complications related to chronic kidney disease (CKD) have not been resolved, including cardiovascular disease (CVD), mineral and bone disorders (CKD-MBD), and infection [1]. Nephrology is a relatively new sub-specialty in the field of internal medicine, and we are still learning the extent of how the kidneys support the body. The social and economic burdens of dialysis are growing worldwide as the number of patients increases. Dialysis is becoming a heavy burden even in developed countries. Thus, preventing end-stage kidney disease (ESKD) is of the utmost importance. Early detection and treatment is recommended because late referral is common, with most CKD patients remaining asymptomatic until a late stage. According to the annual report from the Japanese Society for Dialysis Therapy (JSDT), three-quarters of dialysis patients initiated dialysis therapy within 1 year after referral to the facility [2].

CKD is clinically defined by the presence of albuminuria and/or a decrease in kidney function for >3 months. Since its introduction in 2002, the definition of CKD has been widely accepted not only by nephrologists but also other medical specialties, such as cardiologists and general practitioners. Japan has a long history of universal screening for school children, university students, and employees of companies and government offices. The urine test for proteinuria and hematuria is popular among Japanese people; however, the outcomes have not been well studied.

## Okinawa dialysis study (OKIDS)

**Fig. 1:**
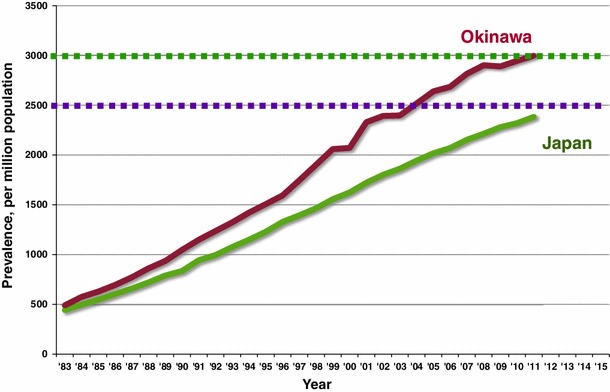
Prevalence of chronic dialysis patients per million population in Okinawa and Japan (cited from ref. [2])

Chronic dialysis therapy was started in Okinawa in 1971, several years after it was initiated in other parts of Japan [3–5]. The number of dialysis patients per million population (pmp) is increasing faster in Okinawa than the national average (Fig. 1). The number was 1,982 in 1990 and 5,246 in 2000 when the population was 1.2 million (1990) and 1.3 million (2000), respectively. The number of dialysis units was 27 in 1990 and 56 in 2000. Initially, the objective of the OKIDS was to determine the relative risk of CVD, including stroke and acute myocardial infarction, in dialysis patients. The strengths of the study are that all of the medical facilities have cooperated, and the data for the incidence of CVD in the general population were available at the same time in Okinawa. We found that the relative risk of stroke, in particular cerebral hemorrhage was very high, but not as high as acute myocardial infarction. The incidence of cerebral hemorrhage was higher than in the general population, even for normotensive dialysis patients [6, 7].

We examined the effects of clinical and laboratory data from several sources on survival [8–18]. Among them, serum albumin was a strong predictor of death, suggesting the importance of nutritional management [9]. Heart failure has been the leading cause of death among dialysis patients. Our data suggest that factors other than atherosclerotic heart disease lead to heart failure in the dialysis population. The overall survival was higher for those with a higher blood pressure and total serum cholesterol, which contradicts data from the general population. These observations were later recognized as ‘reverse epidemiology’ [19]. Dialysis patients have multiple modifiable risk factors. Table 1 summarizes the factors related to poor survival in chronic dialysis patients [20].

**Table 1:** Risk factors for death in chronic dialysis patients (modified from Iseki et al. CEN2004 [20])

Patient demographics
Age
Sex
Primary renal disease (diabetes, nephrosclerosis)
Predialysis comorbid conditions (cardiovascular disease, malignancies)
Laboratory variables
Hypertension
Hypotension
Hypoalbuminemia
Hypocholesterolemia
High CRP
High coronary artery calcification score
CKD-MBD
Hyper- and hypophosphatemia
Hypercalcemia
Electrolyte disturbance
Hyperpotassemia
Hyponatremia

Several randomized controlled trials, such as the treatment of anemia using an erythropoietin-stimulating agent [21, 22] and statin treatment [23, 24], have failed to show an improvement in survival. Hypertension is a major risk factor for death and cardiovascular disease in dialysis patients, but the effect of lowering blood pressure in this high-risk patient group is uncertain. We examined the effect of an angiotensin receptor blocker on survival [25]. In a multicenter prospective, randomized, open-label, blinded-endpoint trial, we assigned 469 patients on chronic hemodialysis (HD) with hypertension to receive the angiotensin receptor blocker olmesartan (*n* = 235) or a treatment other than an angiotensin receptor blocker or angiotensin-converting enzyme inhibitor (*n* = 234). Lowering blood pressure with an angiotensin receptor blocker did not significantly lower the risk of major cardiovascular events or death among patients with hypertension on chronic HD [26].

Two community-based registries for ESKD patients and general screening have been available to us [27, 28]. The Okinawa General Health Maintenance Association (OGHMA) has been performing universal screening annually in Okinawa. Since 1983, they have filed records in the computer registry. With full collaboration of the physicians and medical staff, we were able to match subjects who participated in the screening and later developed ESKD. Because the area consists of sub-tropical islands, the ESKD or CKD stage 5 patients reside exclusively in Okinawa. After verifying the databases from 1983 (*n* = 106,182) and 1993 (*n* = 143,948), we analyzed the relationship between commonly measured laboratory variables and ESKD [27–40]. The total number of identified ESKD patients was 420 from 1983 to 2000.

**Fig. 2:**
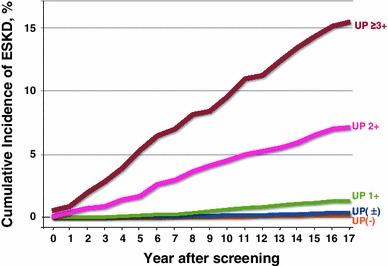
Risk of developing ESKD based on dipstick proteinuria (cited from ref. [28])

Among the variables examined, dipstick proteinuria had the strongest association; the greater the dipstick proteinuria, the higher the risk of developing ESKD (Fig. 2) [28]. Although the dipstick test is ‘semi-quantitative’, the test is clearly ‘dose-dependent’. Serum creatinine was tested in 14 % of patients in 1983 and 35 % in 1993. In addition to dipstick proteinuria, hematuria, blood pressure, body mass index (BMI), serum creatinine, uric acid, and anemia were significant predictors of developing ESKD. Other factors, such as smoking, plasma glucose, dyslipidemia, and metabolic syndrome, also played a role in the development of CKD and ESKD, suggesting the necessity of a multidisciplinary approach. The risk factors related to the development of ESKD are summarized in Table 2 [41].

**Table 2:** Risk factors for the development of ESKD (modified from Iseki et al. CEN2005 [41])

Patient demographics
Age
Sex
Race
Past history of cardiovascular disease
Family history of cardiovascular disease
Clinical and laboratory variables
Proteinuria
Hematuria
Hypertension
Diabetes (hyperglycemia)
Hyperuricemia
Anemia
Low eGFR
Lifestyle
Smoking
Obesity, metabolic syndrome
Sleep disturbance

Only a few studies outside Japan have examined the effect of microhematuria on developing ESKD. Microhematuria is relatively common, particular in elderly women. Compared to proteinuria, the risk of microhematuria was significant, but showed a weak dose–response relationship. The absolute risk of microhematuria was low but was a statistically significant predictor of ESKD [42]. Notably, microhematuria is a risk factor for developing proteinuria; if combined with proteinuria, the risk of developing ESKD is even higher compared to having proteinuria alone [43].

## The Japanese Society for Dialysis Therapy (JSDT)

**Fig. 3:**
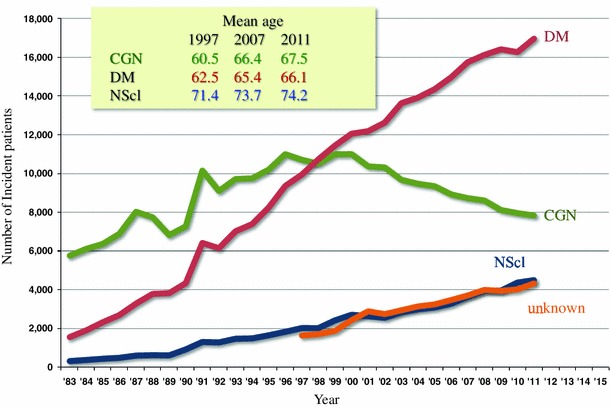
Causes of primary kidney disease among hemodialysis patients in Japan (cited from ref. [2])

**Fig. 4:**
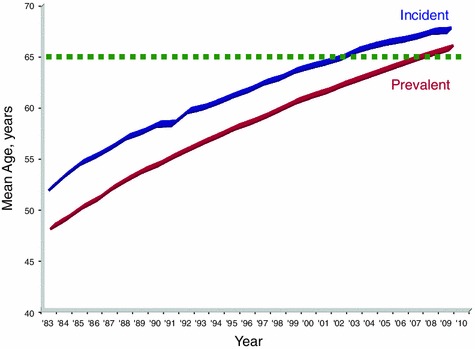
Mean age of chronic dialysis patients in Japan (cited from ref. [2])

The JSDT has been conducting a nationwide survey on chronic dialysis therapy and reporting annually as ‘an overview of regular dialysis treatment in Japan’. According to the 2011 report, the total number of dialysis patients was 304,592 (2,383 pmp), and the leading cause of ESKD was diabetes (44.2 %) (Fig. 3) [2]. The mean age has increased steadily and was 67.8 years in incident and 66.5 years in prevalent patients (Fig. 4). This result is most likely explained by the delay in CKD progression and better survival among the Japanese. The number of patients with chronic glomerulonephritis has decreased linearly since 1998, and the mean age at the start of dialysis has increased from 60.5 years in 1997 to 67.5 years in 2011.

**Fig. 5:**
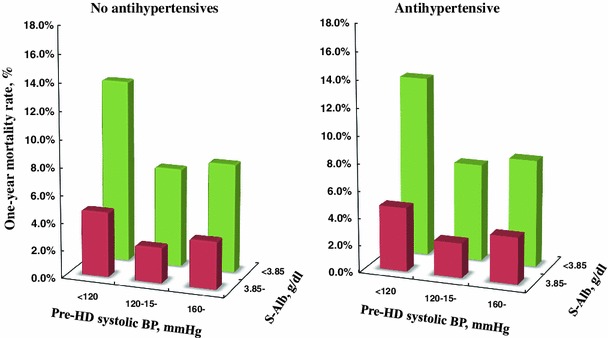
Annual mortality rate of dialysis patients based on pre-hemodialysis blood pressure and serum albumin (cited from ref. [44])

Since 1983, the outcomes of dialysis patients have been investigated. As shown in the OKIDS data, hypoalbuminemia is a significant predictor of death regardless of the pre-dialysis blood pressure and use of anti-hypertensive drugs (Fig. 5) [44]. Survival among Japanese dialysis patients is better than patients in Europe and the United States, yet the reasons for this difference remain to be determined. The demographics and practice patterns differ in several ways. Patient compliance among Japanese patients to a dialysis regimen is good. The most common vascular access is an arteriovenous fistula. A relatively small body size, with a mean BMI of approximately 21 kg/m^2^, might be advantageous for receiving adequate dialysis. Renal transplantation is performed in approximately 1,000–1,200 patients, and cadaveric donation is stable at approximately 200 annually.

The early initiation of dialysis has been practiced worldwide, and the mean initial estimated glomerular filtration rate (eGFR) is becoming higher than ever before [45–47]. The eGFR threshold for starting dialysis is not available. According to the JSDT, the survival was best at around eGFR 4–6 ml/min/1.73 m^2^ [48, 49]. The effect of confounding variables other than age and diabetes is unknown, and we need more data to determine the eGFR threshold. Most Japanese nephrologists rely on the research group criteria supported by the Ministry of Health, Welfare, and Labor, which use eGFR and the presence of uremic symptoms. The threshold for manifesting ‘uremic symptoms’ is variable between patients. Judging the ‘right timing’ would be determined ideally by the physician, patient, and family. Continuing conservative management without dialysis is an alternative option for elderly patients.

## The Japanese Society of Nephrology (JSN)

The JSN has published the ‘Clinical Practice Guidebook for Diagnosis and Treatment of Chronic Kidney Disease’ in 2007, 2009, and 2012 [50]. The “Evidence-based Practice Guideline for the treatment of CKD” was published in 2009 and will be updated in 2013 [51]. The JSN has been raising awareness of CKD on World Kidney Day, which is on the second Thursday in March. Importantly, Japanese patients generally have a lower eGFR compared to American patients. Therefore, an eGFR ≥60 ml/min/1.73 m^2^ is considered to be normal for someone who is otherwise healthy. Albuminuria can only be measured and reimbursed for patients with early-stage diabetic kidney disease in Japan. Instead, the JSN advocates using dipstick proteinuria or measuring the daily amount of proteinuria.

The JSN has been supporting the research project ‘Frontier of Renal Outcome Modifications in Japan’ (FROM-J) [52]. To prevent or halt CKD and ESKD, general practitioners and medical staff, such as dieticians and public health nurses, must be involved. The JSN referral criteria for nephrologists were published to facilitate comprehensive care for CKD patients (Table 3) [50]. Additionally, the Asian Forum of CKD Initiatives (AFCKDI) was started to exchange information on CKD at the inaugural 50th JSN meeting in Hamamatsu in 2007.

**Table 3:** JSN criteria for referring CKD patients to a nephrologist (cited from ref. [50])

Proteinuria (≥2+ by dipstick proteinuria)
Combined proteinuria and hematuria (both 1+ and over by dipstick proteinuria)
Low eGFR (<50 ml/min/1.73 m^2^): <60 ml/min/1.73 m^2^ (if age <40 years) and <40 ml/min/1.73 m^2^ (if age ≥70 years)

**Fig. 6:**
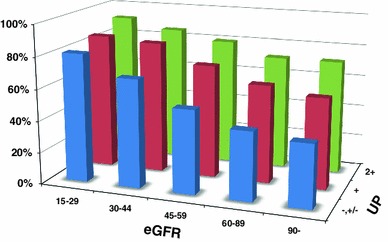
Prevalence of hypertension based on the new KDIGO CKD classification (cited from ref. [53])

Since 2008, the special health check system (so-called Tokutei-Kenshin) has been used to detect subjects with metabolic syndrome and direct them towards a healthy lifestyle. The target population is the 40–74 year age group. The new ‘Kidney Disease: Global Outcomes Improving Outcomes’ (KDIGO) CKD classification prevalence of hypertension was clearly dependent on eGFR and proteinuria (Fig. 6) [53]. Similarly, the prevalence of CVD was dependent on both eGFR and proteinuria. Thus, the JSN is negotiating for a better screening system for CKD in Japan. The JSN has launched web-based registries for CKD and kidney biopsy recipients [54, 55]. Several other research projects are currently being conducted.

## Kidney Disease: Global Outcomes Improving Outcomes

Since the introduction of the concept of CKD, the definition has been challenged with several criticisms: (1) the classification was too simple, (2) lack of key outcomes of CKD, and (3) significance of testing eGFR and albuminuria. In this setting, the KDIGO-Controversies Conference was held from October 4–6, 2009 in London [56]. We offered the dataset, including serum creatinine and dipstick proteinuria, for the conference. After the conference, the CKD classification was slightly modified and expressed as ‘the CKD heat map’. The clinical impacts of eGFR and albuminuria were investigated for several major outcomes [57–61].

To further examine the significance of the classification, the KDIGO CKD prognosis consortium (PC) was organized. We are privileged that the Okinawa 1983/1993 cohorts were involved in the KDIGO-PC. The phase 2 analyses have already been completed for seven major topics, such as hypertension, diabetes, gender, ethnicity, age, CKD epidemiology collaboration, and cystatin C [62–64]. The significance of a low eGFR and albuminuria was confirmed for all-cause mortality and cardiovascular mortality. The relative risks of these markers were similar, but the absolute risks were different based on age, sex, and the presence of diabetes or hypertension. Currently, there will be an additional 13 topics in the Phase 3 step to be studied soon. The new KDIGO ‘Clinical Practice Guideline’ will be published shortly [65].

## Summary

**Fig. 7:**
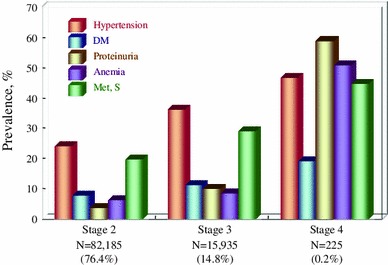
Complications by baseline eGFR among the screened population (unpublished observation)

CKD is common but treatable if detected early and properly managed. At an early CKD stage, patients are usually asymptomatic; therefore, regular health checks using a urine dipstick and serum creatinine are recommended. The intervals for follow-up, however, are debatable due to the cost. In this regard, subjects with hypertension, diabetes, anemia, and/or metabolic syndrome have the highest risk of CKD (Fig. 7). Other factors, such as dyslipidemia, hyperuricemia, gout, CVD and/or a family history of CKD or ESKD, also have a high risk for CKD. Such people should have serum creatinine and albuminuria (proteinuria) assessed at least annually.

CKD patients are at risk of developing acute kidney injury due to contrast media, nephrotoxic drugs, surgery, and dehydration. CKD is a strong risk factor for developing CVD and death and also plays an important role in infection and malignancies, particularly in elderly people. People can live longer with healthy kidneys.

## Personal perspective

Japan is a front runner in ‘the new society’ of a world where the elderly population (≥65 years) is the most prevalent, reaching 30 % in 2020 [66]. Moreover, the total population is decreasing. Japan is the leader of medicine for an aged society and the science of ageing. We need further studies on the natural history of CKD progression and GFR trajectory [67]. High-quality observational studies could promote basic science and stimulate the invention of new treatments for CKD. The mechanisms of age-related GFR decline are entirely unknown, and we have no way to delay the process. Further research on the role of CKD along with other medical conditions, such as infection, mental disorders, CKD-MBD, and malignancies is needed, especially among the elderly population. CKD campaigns in public and medical communities should be continued in order to delay, if not prevent, the development of ESKD. Many cases of CKD are left unrecognized, but the condition can be treated even at late stages, so screening is always beneficial.
